# Supplementary material for: Controllable Doping Characteristics for WSxSey Monolayers Based on the Tunable S/Se Ratio
Source: Nanomaterials (Basel). 2023 Jul 19;13(14):2107. doi: 10.3390/nano13142107 (PMC10385163; doi:10.3390/nano13142107)
Supplement: Supplementary file 1 [file nanomaterials-13-02107-s001.zip › nanomaterials-2481666-supplementary.pdf]

nanomaterials

Supplementary Materials

Controllable Doping Characteristics for WS_xSe_y Monolayers Based on the Tunable S/Se Ratio

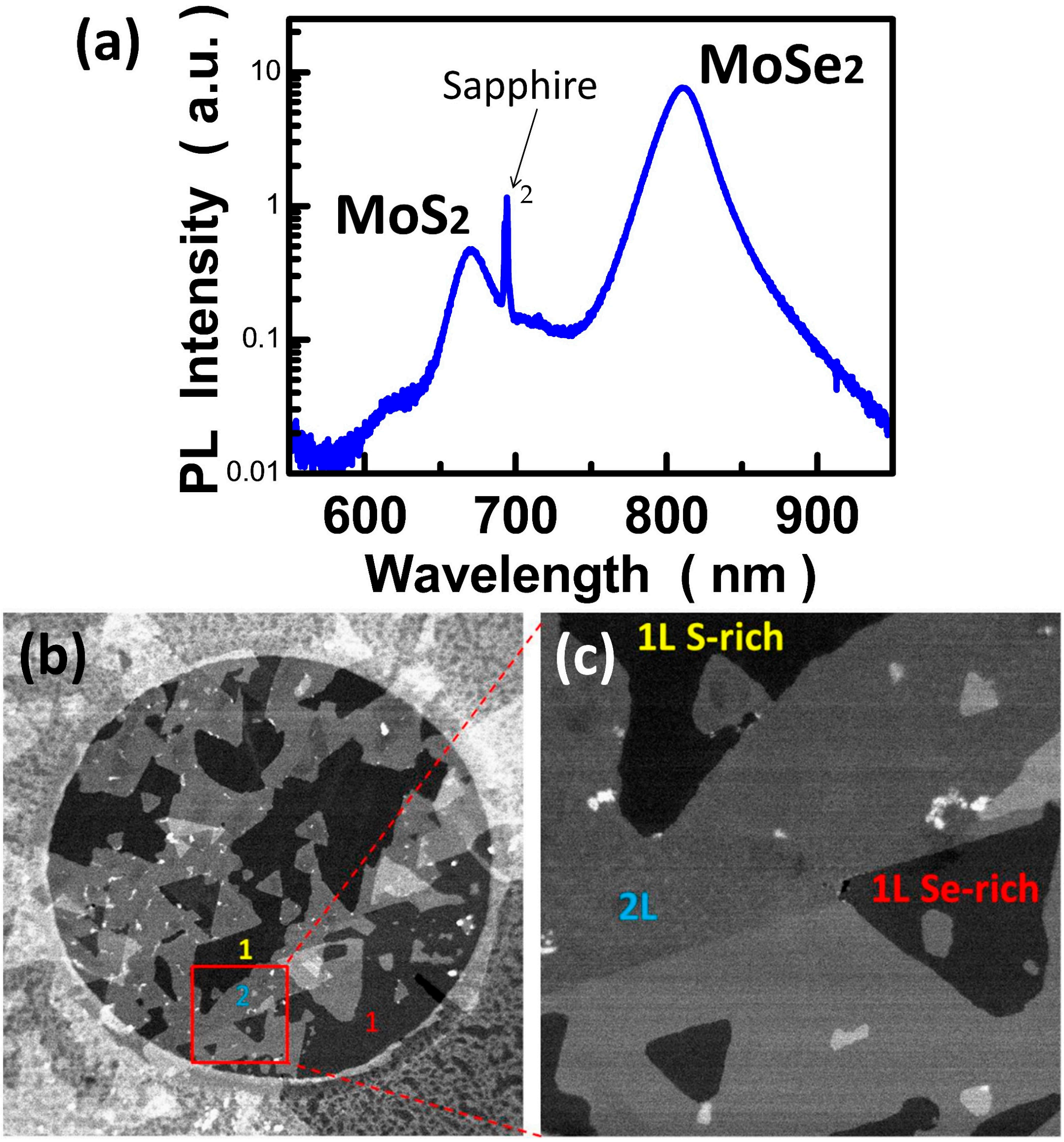

Figure S1. (a) PL spectrum showing the two characteristic peaks belonging to MoS_2 and $MoSe_2$ respectively. (b,c) The bright-field TEM images showing the distinguishable S- and Se-rich parts in the WS_xSe_y.

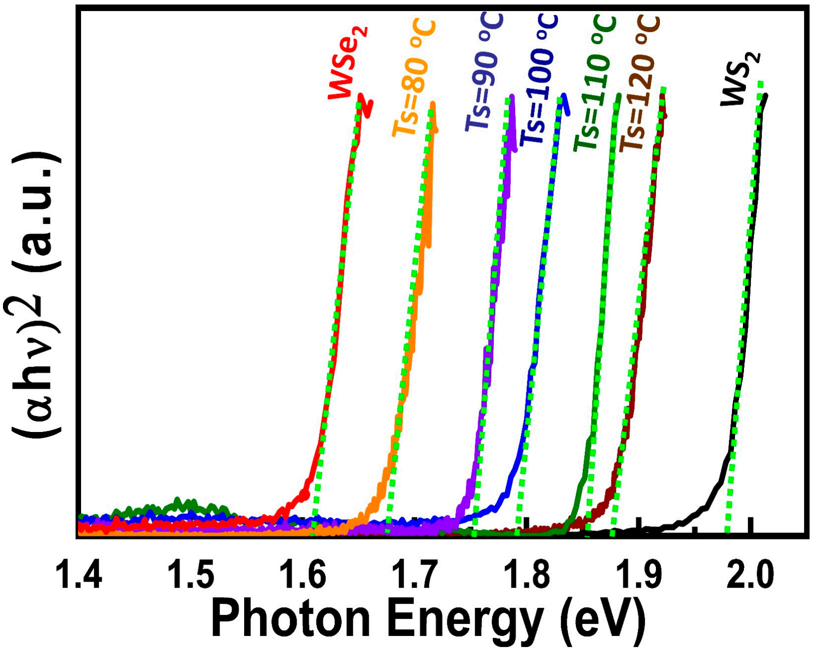

Figure S2. Plots of the $(\alpha h\nu)^2$ versus photon energy ($h\nu$) for direct inter-band transitions of WS_2, WS_xSe_y, and WSe_2 monolayers whose intercepts of tangents represent the band gap energy.

The optical Eg based on direct inter band transitions can be derived from the absorption coefficient equation as followed: $\alpha = B_d(h\nu - Eg)^{1/2}/h\nu$, where α is the optical absorption coefficient, B_d is the absorption constant, h is the Planck constant and ν is the frequency of the incident photon. Figures S2 show plots of $(\alpha h\nu)^2$ versus the photon energy ($h\nu$) for the WS_2, WS_xSe_y at Ts=120, 110, 100, 90 to 80 °C, and WSe_2, where the linear fit intercepts representing the optical Eg are 1.97, 1.88, 1.85, 1.79, 1.75, 1.67, and 1.61 eV, respectively.

Table S1. The chemical stoichiometries for the WS_2, WS_xSe_y at Ts=120, 110, 100, 90 to 80 °C, and WSe_2 monolayers.

		WS_2	Ts=120℃	Ts=110℃	Ts=100℃	Ts=90℃	Ts=80℃	WSe_2
ratio	W	1	1	1	1	1	1	1
	S	2.20	1.87	1.66	1.54	1.12	0.88	0
	Se	0	0.31	0.40	0.48	1.00	1.39	1.77

Table S2. The band gap (E_g), work function (ϕ), and E_F-E_V for the WS_2, WS_xSe_y at Ts=120, 110, 100, 90 to 80 °C, and WSe_2 monolayers.

	WS_2	Ts=120℃	Ts=110℃	Ts=100℃	Ts=90℃	Ts=80℃	WSe_2
Bandgap energy (eV)	1.97	1.88	1.85	1.79	1.75	1.67	1.61
Work function (eV)	4.31	4.17	4.06	4.03	4	3.96	3.95
E_F-E_V (eV)	0.735	0.835	0.885	0.90	0.91	0.91	0.96
